# Progression rate of ankylosing spondylitis in patients with undifferentiated spondyloarthritis: A systematic review and meta-analysis

**DOI:** 10.1097/MD.0000000000005960

**Published:** 2017-01-27

**Authors:** Qing Xia, Dazhi Fan, Xiao Yang, Xiaona Li, Xu Zhang, Mengmeng Wang, Shengqian Xu, Faming Pan

**Affiliations:** a Department of Epidemiology and Biostatistics, School of Public Health, Anhui Medical University, Hefei, Anhui; b Department of Obstetrics, South Medical University Affiliated Maternal and Child Health Hospital of Foshan, Foshan, Guangdong; c Department of Rheumatism and Immunity, the First Affiliated Hospital of Anhui Medical University, Hefei, Anhui, China.

**Keywords:** ankylosing spondylitis, disease progression, follow-up study, undifferentiated spondyloarthritis

## Abstract

**Background:**

The idea that undifferentiated spondyloarthritis (uSpA) represents the early undifferentiated stage of ankylosing spondylitis (AS) and other well-defined SpA subtypes is well known. The gist of this study is to assess the rate estimate of patients with uSpA evolved to AS during long-term follow-up.

**Methods:**

A systematic search was implemented to identify pertinent articles. The primary outcome was the rate estimate that patients with uSpA fulfilling the diagnosis of AS according to the modified New York criteria during follow-up. The rate estimate and corresponding 95% confidence interval (95%CI) were pooled by the random-effects model in STATA 11.0 software. Meta-regression analyses were adopted to explore the sources of heterogeneity. The quality assessment was conducted by the National Institutes of Health Quality Assessment Tool for Observational Cohort and Cross-Sectional Studies and the Begg test and the Egger test were applied to assess publication bias.

**Results:**

Sixteen papers were finally included in this study after screening 1299 citations. The pooled rate of patients with uSpA progression to AS synthesized from the 16 papers was 0.323 (95%CI 0.257–0.389). Subgroup analysis based on the length of follow-up showed that the rate at the time-point of 5, 8, and 10 years follow-up was 0.220 (95%CI 0.110–0.330), 0.291 (95%CI 0.257–0.325), and 0.399 (95%CI 0.190–0.608), respectively; while the rate in Asia, Europe, and Latin America was 0.367 (95%CI 0.282–0.452), 0.228 (95%CI 0.066–0.390), and 0.269 (95%CI 0.209–0.329), respectively. Meta-regression analysis indicated that the length of follow-up alone accounts for 45.23% of the total heterogeneity. Nearly half of the papers scored fair quality and none publication bias was identified based on the Begg test and the Egger test. Further, line chart describes an obviously increased trend for the patients with uSpA fulfilling the diagnosis of AS over time.

**Conclusion:**

The progression rate of patients with uSpA evolved into AS was variable in different time-point, this variation can mostly be explained by the length of follow-up. Thus, more studies with similar time point of follow-up are needed to clarify the full spectrum of uSpA.

## Abstract 2

Supplemental Digital Content is available in the text

## 1 Introduction

The definition of “undifferentiated spondyloarthritis (uSpA)” was firstly used by Burns *et al*^[1]^ in 1982 for patients who had symptoms of spondyloarthritis (SpA) but failed to meet the specific criteria for other definite SpA: namely ankylosing spondylitis (AS), psoriatic arthritis (PsA), reactive arthritis, and arthritis related to inflammatory bowel disease. Patients with these diseases have common clinical manifestations, histopathological features, and responses to treatment which suggested that SpA share common etiological features, including genetic predisposition.^[2]^ The absence of specific diagnostic criteria for uSpA makes the identification and treatment of these patients difficult. Although the classification criteria for SpA developed by Amor *et al*^[3]^ and by the European SpA Study Group (ESSG)^[4]^ have been widely applied for several years, it remained to be seen whether they would be effective in identifying uSpA.

The prevalence of uSpA was ranged from 0.03% to 0.10% in European individuals, 0.15% to 0.55% in Asian, and 0.20% to 1.3% in Northern Arctic indigenous people as reported in a recent systematic review aiming to summarize the prevalence of SpA and its subtypes in the general population.^[5]^ The therapies for uSpA mostly come from the experiences that have been gained in the treatment of other SpA, especially in AS and PsA. Nonsteroidal antiinflammatory drugs (NSAIDs), cyclooxygenase-2 inhibitors, and disease-modifying antirheumatic drugs are the main therapeutic agents used in uSpA. Compared to AS, tumor necrosis factor-α (TNF-α) blockers were rarely recommended and adopted in patients with uSpA except the cases that exist axial involvement or have high risk of developing AS.^[6]^

With the development of the Assessment of SpondyloArthritis international Society classification criteria,^[7–9]^ especially the introduction of magnetic resonance imaging, it is possible to identify patients in their early stage and to recognize the full spectrum of SpA during clinical practice. Ahead of the Assessment of SpondyloArthritis international Society classification criteria became available, many studies have conducted to describe long-term behavior of uSpA. The idea that uSpA represents the early undifferentiated stage of AS and other well-defined SpA subtypes is well known.^[10–13]^ However, follow-up studies have shown discrepancies in both the proportion of patients with uSpA who fulfill AS diagnostic criteria and in the disease duration when patients do fulfill AS criteria. After following 115 patients with uSpA for 10 years, Yunxia *et al*^[14]^ showed that 68 patients presented development to AS (59.1%). A total of 36.4% AS patients was confirmed after 10 years’ follow-up of 88 German uSpA.^[15]^ Although in a Brazilian population-based study, the proportion of AS at the end of 10 years’ follow-up was 24.3%.^[16]^ On top of these, it remains unpredictable whether uSpA patients progress to AS. For example, of the 52 patients in the outcome study, only 4 patients (7.7%) fulfilled the diagnosis of AS at completion of the 5-year period.^[17]^ Due to the inconsistent results reported in different studies, we aim to systematically summarize the rate of patients with uSpA evolved to AS during long-term follow-up, and hope to supply objective evidences to alert clinicians and patients that the probability of patients with uSpA evolved to AS in the certain time-point, fighting for the early identification of these patients.

## 2 Methods

### 2.1 Literature search strategy

This meta-analysis was conducted according to the Preferred Reporting Items for Systematic Reviews and Meta-analyses guidances.^[18]^ Two authors (QX and DF) independently searched the electronic databases including PubMed, Web of Science, EBSCOhost, the Cochrane Library, the Chinese National Knowledge Infrastructure, and Wanfang (Chinese) databases to identify potential studies. The last search was performed on June 25th, 2016 using the following strategy: “undifferentiated spondyloarthritis” or “undifferentiated spondylarthropathy” or “undifferentiated Spondylarthropathies” or “undifferentiated spondyloarthropathy” or “undifferentiated spondyloarthropathies” or “undifferentiated SpA” or “uSpA”, in combined with “ankylosing spondylitis” or “AS.” For publications in Chinese, we used the same combinations of keywords translated into Chinese. The bibliographies of all selected articles and relevant reviews were scrutinized to obtain additional pertinent publications and to elevate the comprehensiveness of the search. Screening of eligible studies was conducted in 2 steps: first, 2 authors (QX and DF) independently reviewed the titles and abstracts of the retrieved studies to identify potentially eligible studies, and then reviewed their full texts. A 3rd reviewer (FP) was consulted for a final decision in case of any discrepancies between the 2 reviewers. All the retrieved references were managed in EndNote X7 (Thomson Reuters).

### 2.2 Inclusion and exclusion criteria

In this study, we only included observational studies directly elaborating the outcomes of patients with uSpA, and each study should contain the progression rate or sufficient information to calculate the progression rate of AS in patients with uSpA (from 1982 to 2016). The enrollment of uSpA was based on the Amor *et al*^[3]^ or ESSG criterion^[4]^ but failed to meet the criteria of AS and other established-SpA. Older studies, where the intention was to recruit uSpA, but which were carried out before the Amor or ESSG criterion, were widely recognized and included. AS was diagnosed according to the modified New York criteria.^[19]^ To be included, the maximum length of the follow-up must longer than 3 years to obtain the long-term outcomes of uSpA in case of the less informative content of shorter studies. Studies fulfilling the following criteria were excluded: duplication of a previous publication; and conference abstracts, reviews, case reports, or editorial studies. No language restrictions were applied.

### 2.3 Data extraction

Two authors (QX and DF) independently performed data extraction using a standardized form. Discrepancies were resolved by consensus, and if agreement could not be reached a 3rd reviewer (FP) was involved to reach consensus. Narrative summaries of the articles were compiled that highlighted the following characteristics: name of first author, year of publication, period of subjects enrollment, country, study location, sample size, male to female ratio (M:F), age of onset, length of follow-up, the number of patients diagnosed with AS at the end of follow-up, diagnostic criteria for enrollment, medicine usage during follow-up, and loss to follow-up. Study location was subdivided into the following categories: Asia, Europe, and Latin America. Diagnostic criteria for enrollment of patients with uSpA mainly contained 3 criteria: Amor criteria, ESSG criteria, and clinical diagnosis (defined by author themselves prior to ESSG criteria and Amor criteria). In present study, we used the maximum time of the follow-up as the length of follow-up duration in each study included. For example, if the time-point (follow-up duration) was recorded as a range (e.g. 3–6 years), the maximum (e.g. 6 years) not the mean or median (e.g. 4.5 years) was used. To avoid statistical dependence in the estimates, if an article reported the progression rate over time, only the most recent estimation was used.

### 2.4 Data synthesis and analysis

The progression rate was calculated by dividing the sample size by the number of diagnosed patients at the end of each follow-up. Stand error was estimated by using the following quotation: sqrt(r∗(1 − r)/N), r indicates the progression rate, while N represents the sample size, namely the total number of patients participated in follow-up. Pooled progression rates and their 95% confidence intervals (CIs) were used to summarize the weighted effect size for each study grouping variable, using the random-effects model, in which the inferences about the mean or variance of effect-size parameters could apply to the universe of studies from which the study sample was obtained (i.e. this model allows the conclusions to be generalized to a wider array of situations).^[20]^ Besides, random-effects model yields the identical results as fixed-effect model in the absence of heterogeneity.^[21]^ Tau-squared (τ^2^) which reflects the magnitude of between-study heterogeneity in a meta-analysis was used to estimate the between-study heterogeneity.^[20]^ We performed subgroup analyses by the length of follow-up and by study location (Asia vs Europe vs Latin America). In addition, we investigated potential sources of heterogeneity by meta-regression analysis.^[22]^ Factors examined in univariate models were year of publication (as a continuous variable), study location (taking Europe as reference category), length of follow-up (as a continuous variable), sample size (as a continuous variable), diagnostic criteria for enrollment (taking clinical diagnosis as reference category), and quality assessment (by comparing studies with poor quality with fair quality studies). Sensitivity analysis was conducted to evaluate the stability of the meta-analysis. When any single study was deleted, the corresponding pooled rates were not substantially altered, suggesting that the results of this meta-analysis are stable. We did an additional analysis that describes the progression rate of AS in patients with uSpA according to the length of follow-up. All statistical analyses were carried out in STATA software (STATA 11.0, StataCorp, College Station, TX) and GraphPad Prism 5.0 software.

### 2.5 Risk of bias

The quality assessment was conducted by the National Institutes of Health Quality Assessment Tool for Observational Cohort and Cross-Sectional Studies,^[23]^ which included the following 14 items: Defined research question; clear study population; >50% participation rate; uniform inclusion and exclusion criteria; sample size justification; exposure of interest measured before outcome; sufficient time frame between exposure and outcome; examination of different levels of exposure in relation to outcome; defined and evenly applied exposure methods; exposure assessed more than once over time; defined and consistently applied outcome measure; blinding of assessors; loss of follow-up <20%; and key potential confounding variables measured and adjusted statistically for impact between exposure and outcome. Each item was given equal weighting. Quality assessment was performed blindly by 2 authors (QX and DF); in case of disagreements, final decision was reached by team consensus. A score of 13 to 14 was good, 9 to 12 was fair, and studies scoring below 9 were deemed to be of poor quality.^[24]^

Concerning the assessment of publication bias, the Begg test^[25]^ and the Egger test^[26]^ statistical tests were adopted; for the interpretation of these 2 tests, statistical significance was defined as *P* < 0.05.

### 2.6 Ethics

Ethical approval was not required for the present meta-analysis.

## 3 Results

### 3.1 Eligible studies

**Figure 1:**
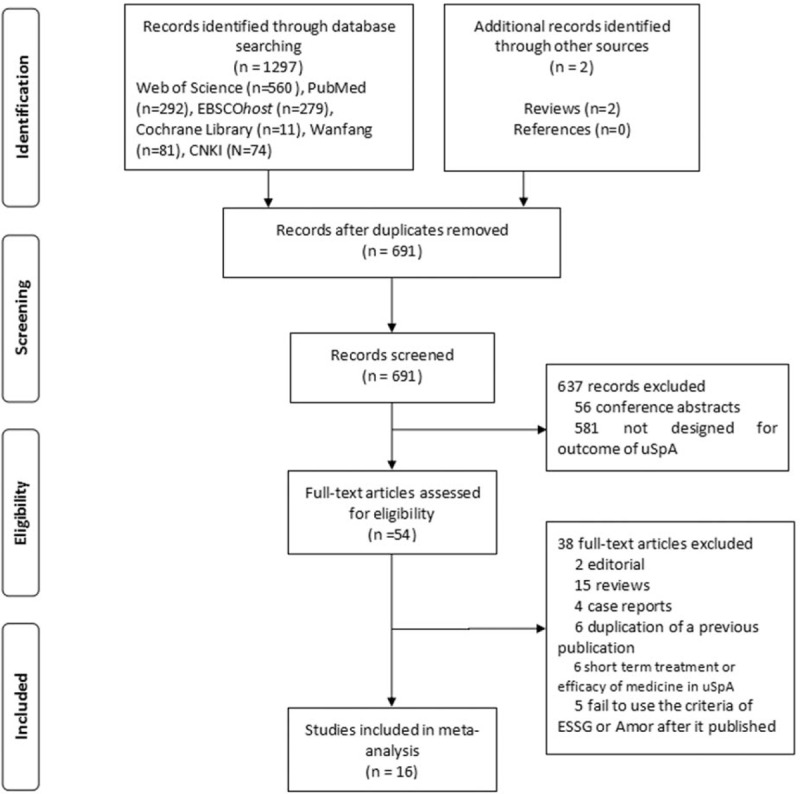
Flow diagram for studies retrieved through the searching and selection processes.

Figure 1 illustrated the process of study selection. A total of 1299 literatures (1297 from databases and 2 from additional sources) were screened by using the predefined search strategy. After duplicates removed, 691 articles were remained. Then 56 conference abstracts and 581 irrelevant studies (not designed for the outcomes of uSpA) were excluded. Fifty-four studies were eligible and needed to be read in full-text. Thirty eight studies were excluded after reading the full-text for the following reasons: 21 were case reports, editorial, and review articles; 6 were short-term treatment or efficacy of medicine in uSpA; 6 were duplication of a previous publication; and 5 fail to use the ESSG criteria or Amor criteria after it published which we reckon as being low in quality. Finally, 16 studies^[14–17,27–38]^ were included in present review.

### 3.2 Study characteristics

The summaries of 16 eligible studies were shown in Table 1 and all studies were prospective. Of the 16 studies included in present study, 3 from Latin America (2 from Brazil^[16,28]^ and 1 from Mexico^[27]^), 3 from Europe (1 from Spain^[17]^ and 2 form Germany^[15,30]^), and 10 from Asia (1 from India^[29]^ and 9 from China^[14,31–38]^). The total number of patients with uSpA participated in follow-up was 2134, with sample sizes ranged from 35 to 648. The age of onset was mainly concentrated in the 3rd decades of individuals. The ratio of male to female (M:F) was range from 0.64:1 to 7.00:1. As shown in Supplemental Table 1, the diagnostic criteria for enrollment of uSpA were based on ESSG criteria in 7 studies, while Amor criteria in 1 study, a combination of ESSG criteria or Amor criteria in 6 studies, and clinical diagnosis in 2 studies. Medicine usages during follow-up were reported in 10 articles, NSAIDs were used for all these patients except 1 study which claimed 7 patients without any treatment. NSAIDs were independently used in patients in 4 articles, while a combination of NSAIDs and disease-modifying antirheumatic drugs was used in the remaining 6 articles.

**Table 1:**
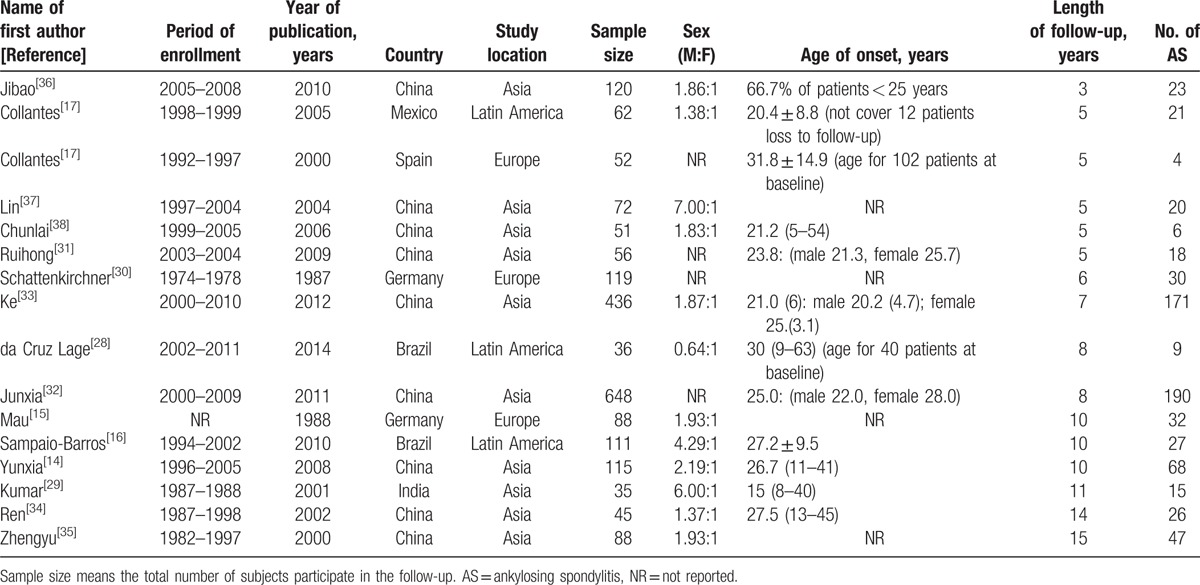
Characteristic of eligible studies.

### 3.3 Rate of AS in patients with uSpA during follow-up

**Figure 2:**
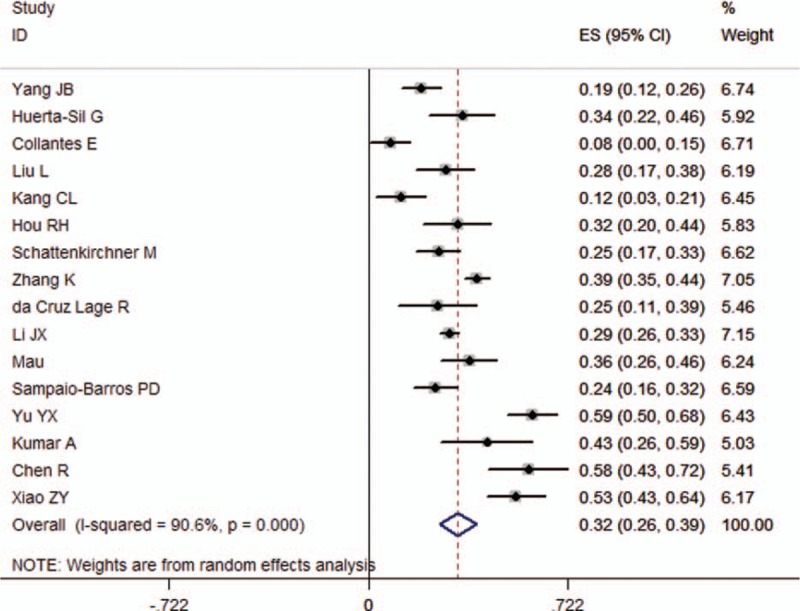
Rate estimates of patients with uSpA evolved to AS (boxes) with 95% confidence limits (horizontal bars) for each study selected; pooled rate estimates are represented as a diamond. AS = ankylosing spondylitis, uSpA = undifferentiated spondyloarthritis.

On the whole, the pooled rate of patients with uSpA progression to AS synthesized from the 16 papers was 0.323 (95%CI 0.257–0.389; τ^2^ = 0.0155) (Table 2; Fig. 2). Since the progression rates maybe vary among the different length of follow-up, we stratified our data based on the duration of follow-up. Five studies with the progression rate of 5 years were conducted, and the summarized rates was 0.220 (95%CI 0.110–0.330; τ^2^ = 0.0131) (Supplemental Fig. 1A). The progression rate of 8 years was 0.291 (95%CI 0.257–0.325; τ^2^ < 0.001), which was synthesized from 2 long-term follow-up studies (Supplemental Fig. 1B). The desired outcomes in 3 follow-up studies up to 10 years were also obtained. The rate of 10 years was 0.399 (95%CI 0.190–0.608; τ^2^ = 0.0319) (Supplemental Fig. 1C). There were also follow-up studies designed for 3, 6, 7, 11, 14, and 15 years; however, no summarized rates can be pooled from these studies for the absence of similar time-point study. The rate of patients with uSpA evolved into AS for 3, 6, 7, 11, 14, and 15 years was 0.192 (95%CI 0.121–0.262; τ^2^ < 0.001), 0.252 (95%CI 0.174–0.330; τ^2^ < 0.001), 0.392 (95% CI 0.346–0.438; τ^2^ < 0.001), 0.429 (95% CI 0.265–0.593; τ^2^ < 0.001), 0.578 (95%CI 0.433–0.722; τ^2^ < 0.001), and 0.543 (95%CI 0.430–0.638; τ^2^ < 0.001), respectively. Further, subgroup analysis based on study location was also carried out (Asia vs Europe vs Latin America). The results were stable across study location. The rate in Asia, Europe, and Latin America was 0.367 (95%CI 0.282–0.452; τ^2^ = 0.0162), 0.228 (95%CI 0.066–0.390; τ^2^ = 0.0186), and 0.269 (95%CI 0.209–0.329; τ^2^ < 0.001), respectively (Supplemental Fig. 2).

**Table 2:**
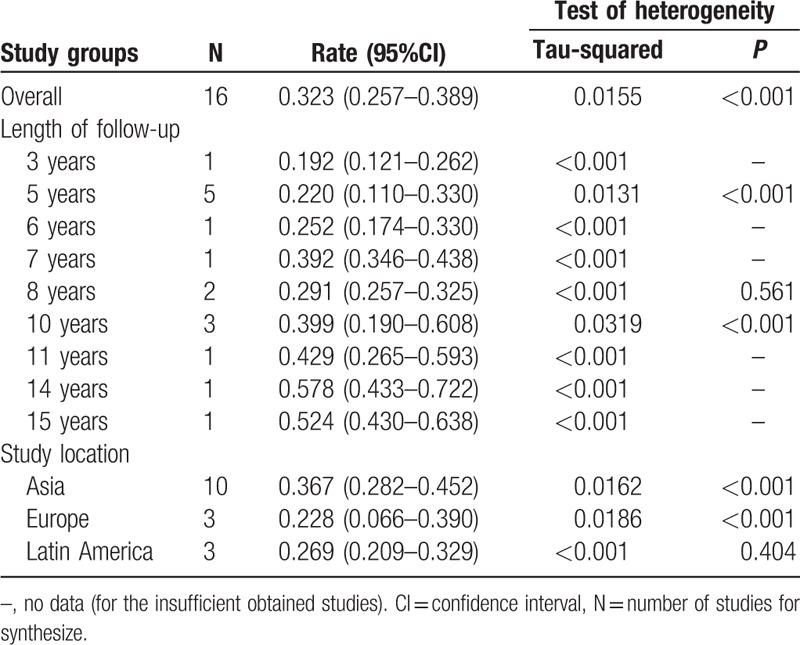
Results of meta-analysis.

### 3.4 Evaluation of heterogeneity

Significant heterogeneity was detected in pooled rate of patients with uSpA to AS (τ^2^ = 0.0155, *P* < 0.001). Consequently, we conducted meta-regression analysis herein to explore the sources of heterogeneity. Year of publication, study location, length of follow-up, sample size, diagnostic criteria for enrollment, and quality assessment were tested as potential sources of heterogeneity in meta-regression analysis. Table 3 shows the results of the univariate meta-regression analysis. The positive association between the length of follow-up and the rate estimate was reflected upon the findings of the meta-regression analysis (exponentiated coefficient = 1.034, 95%CI 1.016–1.052, rate estimate in increments of 0.033, *P* = 0.001). Of note, the τ^2^ declined from 0.0155 to 0.00849 after the meta-regression analysis, which accounts for 45.23% of the total heterogeneity. On the other hand, the remaining sets of meta-regression analyses did not yield any significant associations.

**Table 3:**
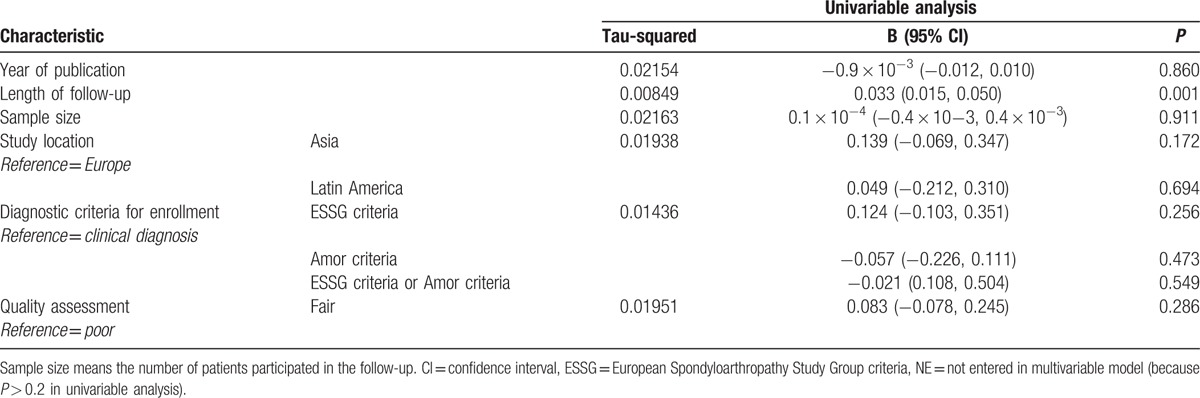
Univariate meta-regression analysis on the progression rate of ankylosing spondylitis.

### 3.5 Evaluation of quality of studies and risk of bias

The evaluation of quality of studies is presented in Supplemental Table 2. The results showed that 7 studies were of fair quality, while the remaining 9 studies were low in quality. The participation rate in Hou *et al*^[31]^ study was lower than 50%, and only 8 studies assessed the rate estimate more than once over time. Loss to follow-up was occurred in 8 studies, and the proportion was larger than 20% in 4 of these studies (details in Supplemental Table 1). None of these studies reported whether the blind method was used or not. There was no evidence of publication bias based on the Begg test (z = 1.44, *P* = 0.150) and the Egger test (t = 0.52, *P* = 0.610).

### 3.6 Sensitivity analysis

Sensitivity analysis was conducted to assess the robustness of the meta-analysis results by removing 1 single study in sequence. No significant change of the pooled rates was found, which indicated the stability of our results (Supplemental Fig. 3).

### 3.7 Rate of AS in patients with uSpA in each time-point

**Figure 3:**
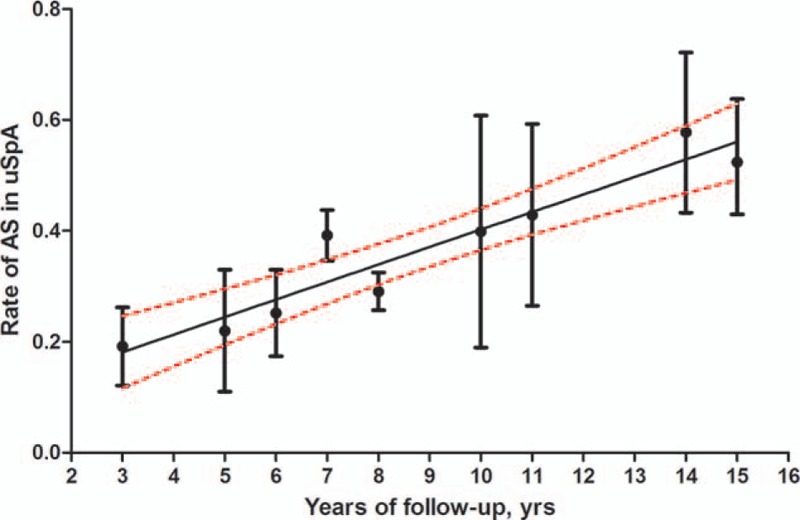
Rate estimates of patients with uSpA evolved to AS (circles) with 95% confidence limits (vertical bars) in each time-point; the black line represents the general trend of rate estimates of patients with uSpA progression to AS developed over time with 95% confidence curves (red lines). AS = ankylosing spondylitis, uSpA = undifferentiated spondyloarthritis.

Using the data above calculated, we furtherly plotted the rate of AS in patients with uSpA in each time-point. As shown in Fig. 3, line chart describes the rate of AS in uSpA increased with the increasing length of follow-up.

## 4 Discussion

USpA accounts for a significant but variable proportion of patients with SpA in different regions across the whole world.^[5]^ The natural course of uSpA is variable. Mounts of reviews regarded uSpA as an early form of AS, and declaimed that the identification of these patients could enable physicians to recognize patients with AS at an earlier stage and treat them accordingly.^[10,11,13]^ Early diagnosis of AS seemed less urgent for many physicians because of the lack of therapeutic options. But now, TNF-α blockers obtain an impressive effect to treatment and could stop disease progression.^[39,40]^ Thus, knowing how many patients with uSpA will progression to AS is of importance to clinical physicians. In present study, we found that 22.0% (95%CI 11.0%–33.0%) patients with uSpA evolved to AS after 5 years’ follow-up, and 29.1% (95%CI 25.7%–32.5%) and 39.9% (95%CI 19.0%–60.8%) were found after 8 and 10 years, respectively. What is more, an obviously increased trend for the patients with uSpA fulfilling the diagnosis of AS over time was identified.

USpA was first recognized in patients with possible AS or early SpA without radiologic evidence of sacroiliitis. Although the diagnosis of “possible AS,”^[15]^ and “HLA-B27 positive oligoarthritis”^[30]^ were not based on Amor criteria and ESSG criteria, previous reviews^[11–13]^ have taken them into account when involved in the development of uSpA. Consequently, these 2 older articles were also included in present systematic review to obtain a comprehensive assessment of the outcomes. In our studies, we only selected studies that elaborated the progression outcomes of patients with uSpA, which should contain sufficient data to calculate the rate of AS in uSpA. In addition, we only enrolled studies with a follow-up longer than 3 years to obtain the long-term outcomes of uSpA, which may lead a small amount of relevant literatures been excluded from present study.

This study is the first that pooled progression rate of AS in patients with uSpA according to the length of follow-up. Univariate meta-regression analysis showed that the length of follow-up maybe the most important characteristics explaining heterogeneity in rate estimates of AS, with the τ^2^ declined from 0.0155 to 0.00849, which accounts for 45.23% of the total heterogeneity. Due to the highly heterogeneity, caution is required when using the pooled result. However, we considered it plausible when refers to the progression rate of AS in uSpA at the time-point of 5, 8, and 10 years. The rate of 5 years’ follow-up was relatively low, and it may be caused from a Spanish study with a definitely low rate.^[17]^ The progression rate of 8 years was robust because of the low heterogeneity. Irrespective of the discrepancy of the number of subjects between the 2 studies,^[28,32]^ the enrollment of study subjects in these 2 studies was almost synchronous, which may improve the rationality of data merging. Higher heterogeneity was detected in the rate of 10 years, and it maybe accounts for the 3 studies^[14–16]^ which come from different regions. Further, a relatively high pooled progression rate of AS was found in Asia by comparing that in Europe and Latin America; however, the estimates came from 4 studies with longest follow-up time in present study.^[14]^

In 2011, De La Mata *et al*^[41]^ conducted a systematic literature review on current evidence of the management of uSpA, and found that TNF-α blockers are beneficial to active uSpA patients, at least in the short term. In a subgroup analysis of patients naive to anti-TNF agents, Paramarta *et al*^[42]^ found that the disease activity index of patients with uSpA remained equivalent to those of AS while higher than PsA, which provide more evidence that uSpA may represent the earliest form of specific SpA, in particular AS. However, biological agent was used in none of the 16 follow-up studies included in present meta-analysis. This phenomenon suggests that uSpA might be inadequately treated in past 3 decades, and subsequently made the progression rate overestimated. It is to be expected that better therapy of uSpA would likely further decrease the progression rate. On the other hand, whether TNF-α inhibitors limit the development of new radiographic lesions is a matter of debate.^[43]^

Some limitations of the present study should be addressed. First, the rate of loss to follow-up >20% was occurred in 4 studies; therefore, selection bias cannot be excluded. In addition, few studies with similar time-point were obtained to calculate the pooled data; thus, we only reported the pooled rate of 5, 8, and 10 years in term of the length of follow-up. On top of these, heterogeneity was detected in present study, which may hamper the generalization of the results.

## 5 Conclusion

This systematic review with meta-analysis summarized the progression rate of AS in patients with uSpA and the large variation in the progression rate of AS is explained by the length of follow-up; thus, similar time-point of follow-up studies are needed to estimate the progression rate of AS in patients with uSpA. Further, medical efficacy, especially biological agents, should be taken into account.

## Acknowledgments

The authors thank to all the staff who participated in this article. The authors also thank grants from the National Natural Science Foundation of China (30972530, 81273169, 81573218, and 81571572) for the support.

## Supplementary Material

Supplemental Digital Content

## References

[1] Burns T Marder A Becks E Undifferentiated spondylarthritis: a nosological missing link? Arthritis Rheum 1982;25 (Suppl):S142.

[2] Brown MA Kenna T Wordsworth BP Genetics of ankylosing spondylitis–insights into pathogenesis. Nat Rev Rheumatol 2016;12:81–91. 26439405 10.1038/nrrheum.2015.133

[3] Amor B Dougados M Mijiyawa M Criteria of the classification of spondylarthropathies. Rev Rhum Mal Osteoartic 1990;57:85–9. 2181618

[4] Dougados M van der Linden S Juhlin R The European Spondylarthropathy Study Group preliminary criteria for the classification of spondylarthropathy. Arthritis Rheum 1991;34:1218–27. 1930310 10.1002/art.1780341003

[5] Stolwijk C van Onna M Boonen A Global prevalence of spondyloarthritis: a systematic review and meta-regression analysis. Arthritis Care Res 2016;68:1320–31. 10.1002/acr.22831 26713432

[6] Cruzat V Cuchacovich R Espinoza LR Undifferentiated spondyloarthritis: recent clinical and therapeutic advances. Curr Rheumatol Rep 2010;12:311–7. 20632136 10.1007/s11926-010-0115-0

[7] Rudwaleit M Landewe R van der Heijde D The development of Assessment of SpondyloArthritis International Society classification criteria for axial spondyloarthritis (part I): classification of paper patients by expert opinion including uncertainty appraisal. Ann Rheum Dis 2009;68:770–6. 19297345 10.1136/ard.2009.108217

[8] Rudwaleit M van der Heijde D Landewe R The development of Assessment of SpondyloArthritis International Society classification criteria for axial spondyloarthritis (part II): validation and final selection. Ann Rheum Dis 2009;68:777–83. 19297344 10.1136/ard.2009.108233

[9] Rudwaleit M van der Heijde D Landewe R The Assessment of SpondyloArthritis International Society classification criteria for peripheral spondyloarthritis and for spondyloarthritis in general. Ann Rheum Dis 2011;70:25–31. 21109520 10.1136/ard.2010.133645

[10] Burgos-Vargas R Spondyloarthritis: from undifferentiated SpA to ankylosing spondylitis. Nat Rev Rheumatol 2013;9:639–41. 24080862 10.1038/nrrheum.2013.146

[11] Burgos-Vargas R Undifferentiated spondyloarthritis: a global perspective. Curr Rheumatol Rep 2007;9:361–6. 17915091 10.1007/s11926-007-0058-2

[12] Zochling J Brandt J Braun J The current concept of spondyloarthritis with special emphasis on undifferentiated spondyloarthritis. Rheumatology (Oxford) 2005;44:1483–91. 16091395 10.1093/rheumatology/kei047

[13] Burgos-Vargas R Casasola-Vargas JC From retrospective analysis of patients with undifferentiated spondyloarthritis (SpA) to analysis of prospective cohorts and detection of axial and peripheral SpA. J Rheumatol 2010;37:1091–5. 20516036 10.3899/jrheum.100413

[14] Yunxia Y Shuhong C Liancao P A ten-year follow up analysis of 115 patients with undifferentiated spondyloarthropathies. Ningxia Med J 2008;30:626–7.

[15] Mau W Zeidler H Mau R Clinical features and prognosis of patients with possible ankylosing spondylitis. Results of a 10-year follow-up. J Rheumatol 1988;15:1109–14. 3262757

[16] Sampaio-Barros PD Bortoluzzo AB Conde RA Undifferentiated spondyloarthritis: a long-term follow-up. J Rheumatol 2010;37:1195–9. 20436080 10.3899/jrheum.090625

[17] Collantes E Veroz R Escudero A Can some cases of ‘possible’ spondyloarthropathy be classified as ‘definite’ or ‘undifferentiated’ spondyloarthropathy? Value of criteria for spondyloarthropathies. Spanish Spondyloarthropathy Study Group. Joint Bone Spine 2000;67:516–20. 11195314 10.1016/s1297-319x(00)00201-3

[18] Moher D Liberati A Fau-Tetzlaff J Preferred reporting items for systematic reviews and meta-analyses: the PRISMA statement. PLoS Med 2009;6:e1000097. 19621072 10.1371/journal.pmed.1000097 PMC2707599

[19] van der Linden S Valkenburg HA Cats A Evaluation of diagnostic criteria for ankylosing spondylitis. A proposal for modification of the New York criteria. Arthritis Rheum 1984;27:361–8. 6231933 10.1002/art.1780270401

[20] Hedges LV Vevea JL Fixed- and random-effects models in meta-analysis. Psychol Methods 1998;3:486–504.

[21] Borenstein M Hedges LV Higgins JP A basic introduction to fixed-effect and random-effects models for meta-analysis. Res Synth Methods 2010;1:97–111. 26061376 10.1002/jrsm.12

[22] Wijnands JM Viechtbauer W Thevissen K Determinants of the prevalence of gout in the general population: a systematic review and meta-regression. Eur J Epidemiol 2015;30:19–33. 25064615 10.1007/s10654-014-9927-y

[23] National Institutes of Health. Quality Assessment Tool for Observational Cohort and Cross-Sectional Studies. National Heart, Lung, and Blood Institute. Avaliable from: www.nhlbi.nih.gov/health-pro/guidelines/in-develop/cardiovascular-risk-reduction/tools/cohort [Accessed November 5, 2015].

[24] Leung A Heal C Perera M A systematic review of patient-related risk factors for catheter-related thrombosis. J Thromb Thrombolysis 2015;40:363–73. 25680892 10.1007/s11239-015-1175-9

[25] Begg CB Mazumdar M Operating characteristics of a rank correlation test for publication bias. Biometrics 1994;50:1088–101. 7786990

[26] Egger M Davey Smith G Schneider M Bias in meta-analysis detected by a simple, graphical test. BMJ 1997;315:629–34. 9310563 10.1136/bmj.315.7109.629 PMC2127453

[27] Huerta-Sil G Casasola-Vargas JC Londono JD Low grade radiographic sacroiliitis as prognostic factor in patients with undifferentiated spondyloarthritis fulfilling diagnostic criteria for ankylosing spondylitis throughout follow up. Ann Rheum Dis 2006;65:642–6. 16219705 10.1136/ard.2005.043471 PMC1798115

[28] da Cruz Lage R de Souza Bomtempo CA Kakehasi AM Undifferentiated spondyloarthritis in a heterogeneous Brazilian population: an eight-year follow-up study. Rheumatol Int 2014;34:1019–23. 23765202 10.1007/s00296-013-2797-x

[29] Kumar A Bansal M Srivastava DN Long-term outcome of undifferentiated spondylarthropathy. Rheumatol Int 2001;20:221–4. 11563579 10.1007/s002960100116

[30] Schattenkirchner M Kruger K Natural course and prognosis of HLA-B27-positive oligoarthritis. Clin Rheumatol 1987;6 (Suppl 2):83–6. 3500826 10.1007/BF02203389

[31] Ruihong H Liyun Z Xiaofeng L A five-year follow up study of 127 patients with undifferentiated spondyloarthropathies. Chin J Allergy Clin Immunol 2009;3:107–11.

[32] Junxia L Liyun Z Ruihong H The follow-up analysis of 1024 patients with undifferentiated spondyloarthropathy. Proc Clin Med 2011;20:3–6.

[33] Ke Z Qian Q Shiwen Y Clinical features and follow-up data of 436 cases of undifferentiated spondyloarthropathies. J Pract Med 2012;28:608–10.

[34] Ren C Qiuqiang L Jingcai X The follow-up analysis of 45 patients with undifferentiated spondyloarthropathy. Chin J Pract Inter Med 2002;22:497–8.

[35] Zhengyu X Qingyu Z Shaobi H A fifteen-year follow up study of undifferentiated spondyloarthropathies. Chin J Rheumatol 2000;4:120–1.

[36] Jibao Y Huiqun Z Zhi L Comparison of undifferentiated spondyloarthropathy patients with positive or negetive HLA-B27. J Bengbu Med Coll 2010;35:252–4.

[37] Lin L Jinbin W Xiangdang W Clinical analysis of 128 patients with undifferentiated spondyloarthropathies. J Med Forum 2004;25:35–6.

[38] Chunlai K Shengnan C Ruihua W Clinical features and prognosis of undifferentiated spondyloarthropathies. Chin Med J Metall Ind 2007;24:57–8.

[39] Brandt J Khariouzov A Listing J Successful short term treatment of patients with severe undifferentiated spondyloarthritis with the anti-tumor necrosis factor-alpha fusion receptor protein etanercept. J Rheumatol 2004;31:531–8. 14994401

[40] Brandt J Haibel H Reddig J Successful short term treatment of severe undifferentiated spondyloarthropathy with the anti-tumor necrosis factor-alpha monoclonal antibody infliximab. J Rheumatol 2002;29:118–22. 11824947

[41] De La Mata J Maese J Martinez JA Current evidence of the management of undifferentiated spondyloarthritis: a systematic literature review. Semin Arthritis Rheum 2011;40:421–9. 429.e421–e423. 20832101 10.1016/j.semarthrit.2010.06.003

[42] Paramarta JE De Rycke L Ambarus CA Undifferentiated spondyloarthritis vs ankylosing spondylitis and psoriatic arthritis: a real-life prospective cohort study of clinical presentation and response to treatment. Rheumatology (Oxford) 2013;52:1873–8. 23861532 10.1093/rheumatology/ket239

[43] Taurog JD Chhabra A Colbert RA Ankylosing spondylitis and axial spondyloarthritis. N Engl J Med 2016;374:2563–74. 27355535 10.1056/NEJMra1406182

