# Supplementary material for: Progression rate of ankylosing spondylitis in patients with undifferentiated spondyloarthritis: A systematic review and meta-analysis
Source: Medicine (Baltimore). 2017 Jan 27;96(4):e5960. doi: 10.1097/MD.0000000000005960 (PMC5287968; doi:10.1097/MD.0000000000005960)
Supplement: Supplemental Digital Content [file medi-96-e5960-s001.doc]

Supplemental Figure 1 Rate estimates of patients with uSpA evolved to AS (boxes) with 95% confidence limits (horizontal bars) for each study selected; pooled rate estimates are represented as a diamond. (A) 5 year follow up, (B) 8 year follow up, and (C) 10 year follow up;


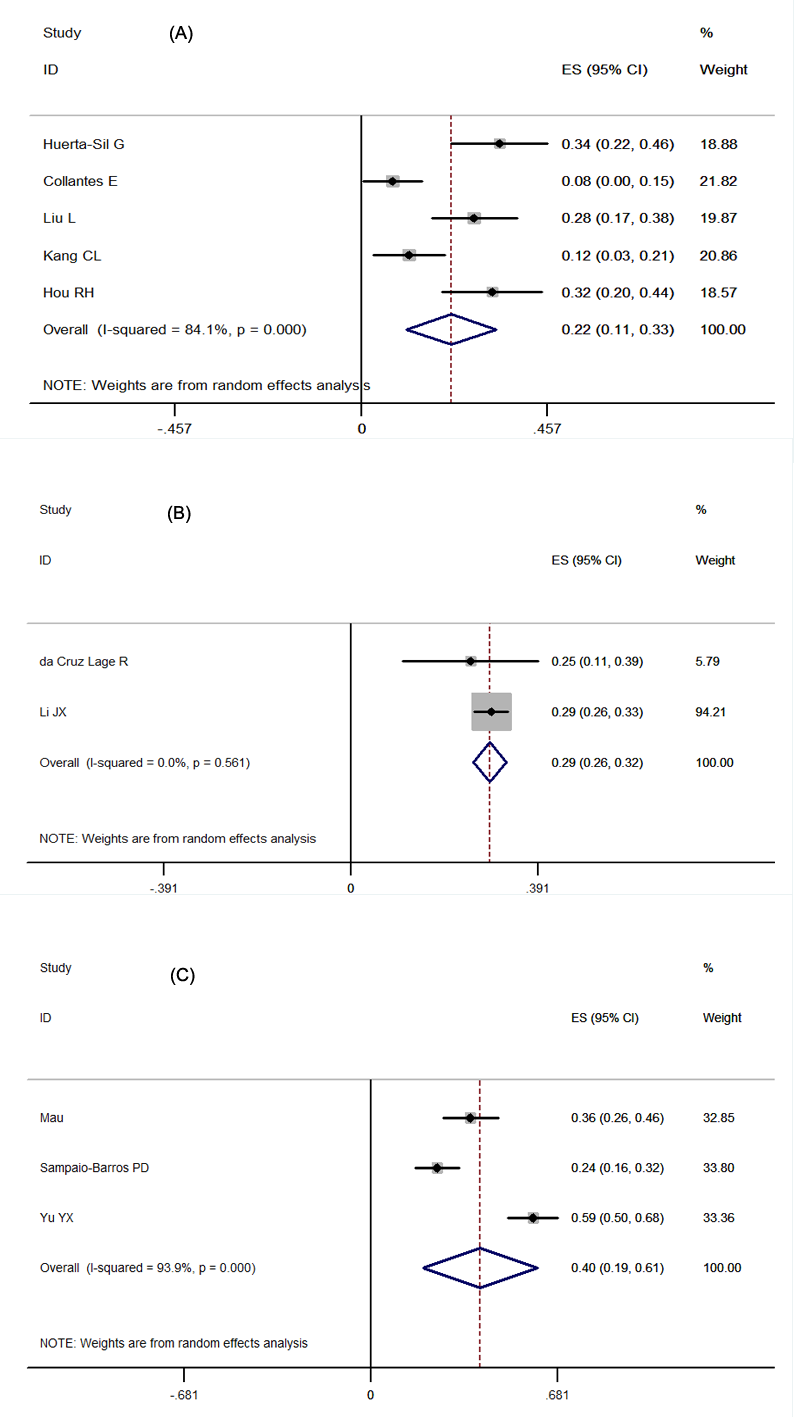


Supplemental Figure 2 Rate estimates of patients with uSpA evolved to AS according to study location;


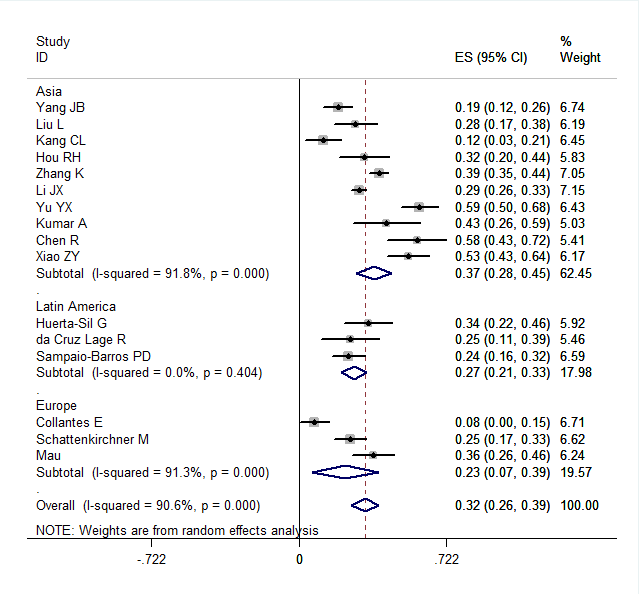


Supplemental Figure 3 Plot of sensitivity analysis.


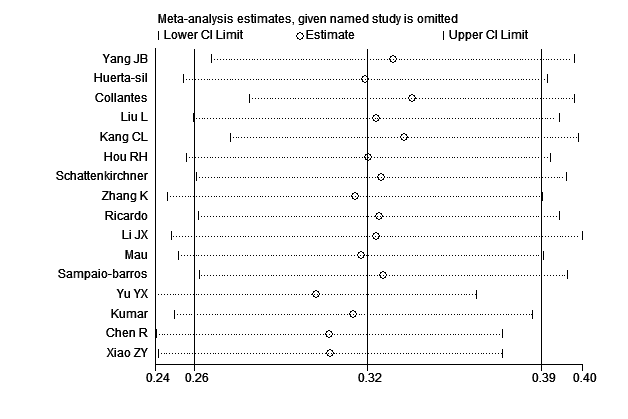


| **Supplemental Table 1.** Diagnostic criteria, medicine usage and loss to follow up in included studies | | | |
| --- | --- | --- | --- |
| **Name of first author** | **Diagnostic criteria for enrollment** | **Medicine usage during follow-up^*^** | **Loss to follow up (n)** |
| Yang JB | Amor’s criteria | NSAIDs combined with SASP and TGP for all | 7 |
| Huerta-Sil G | ESSG criteria | NR | 12 (10 males and 2 females) |
| Collantes E | Amor’s criteria or ESSG criteria | 49% of 102 patients (at baseline) show good response to NSAIDs therapy | 7 |
| Liu L | ESSG criteria | All patients show good response to NSAIDs therapy, NSAIDs combined with SASP and MTX to patients who relapse after the drug withdrawal | NR |
| Kang CL | Amor’s criteria or ESSG criteria | None for 7; NSAIDs for 4; NSAIDs combined with SASP, MTX and TGP for the remaining patients | 18 |
| Hou RH | Amor’s criteria or ESSG criteria | NSAIDs for 8; NSAIDs combined with SASP, MTX and TGP for the remaining patients | 0 |
| Schattenkirchner M | Clinical criteria | NSAIDs for all; corticosteroids for some cases; Chloroquine for 5; gold salts for 2; D-penicillamine for 2 | 5 |
| Zhang K | Amor’s criteria or ESSG | NR | 0 |
| da Cruz Lage R | ESSG criteria | NR | 0 |
| Li JX | Amor’s criteria or ESSG criteria | NSAIDs for 336; NSAIDs combined with SASP, MTX, Chloroquine and TGP for the remaining patients | 0 |
| Mau | Clinical criteria | NR | 34 (4 patients died, 9 moved, 21 refused to participate) |
| Sampaio-Barros PD | Amor’s criteria or ESSG | NSAIDs for all; glucocorticoids for 32 (mainly low doses of prednisone); MTX for 34; SASP for 79 | 69 (2 patients died for reasons not related to SpA, 67 patients were lost at 10 years) |
| Yu YX | ESSG criteria | NSAIDs | 0 |
| Kumar A | ESSG criteria | NSAIDs for all; SASP alone for 7; SASP and MTX for 4; SASP and Chloroquine for 1; SASP and myocrisin for 1; MTX and prednisolone for 1. | 13 |
| Chen R | ESSG criteria | NSAIDs | 0 |
| Xiao ZY | ESSG criteria | NR | NR |
| NSAIDs, non-steroidal anti-inflammatory drugs; ESSG, European Spondyloarthropathy Study Group criteria; NR, not reported; TGP, Total Glucosides of Paeony Capsules; SASP, sulfasalazine; MTX, methotrexate;  **^*^** DMARDs mainly including SASP, CTX, and Chloroquine | | | |

| Supplemental Table 2**.** Quality Assessment Tool for Observational Cohort and Cross-Sectional Studies. | | | | | | | | | | | | | | | | |
| --- | --- | --- | --- | --- | --- | --- | --- | --- | --- | --- | --- | --- | --- | --- | --- | --- |
| Study | Quality assessment point^#^ | | | | | | | | | | | | | | | |
|  | 1 | 2 | 3 | 4 | 5 | 6 | 7 | 8 | 9 | 10 | 11 | 12 | 13 | | 14 | Grade |
| Yang JB | Y | Y | Y | Y | N | NA | Y | NA | Y | N | Y | NR | | Y | N | Poor |
| Huerta-Sil G | Y | Y | Y | Y | N | NA | Y | NA | Y | N | Y | NR | | Y | Y | Fair |
| Collantes E | Y | Y | Y | Y | N | NA | Y | NA | Y | N | Y | NR | | Y | N | Poor |
| Liu L | Y | Y | Y | Y | N | NA | Y | NA | Y | N | Y | NR | | NR | N | Poor |
| Kang CL | Y | Y | Y | Y | N | NA | Y | NA | Y | N | Y | NR | | N | N | Poor |
| Hou RH | Y | Y | N | Y | N | NA | Y | NA | Y | Y | Y | NR | | Y | N | Poor |
| Schattenkirchner M | Y | Y | Y | Y | N | NA | Y | NA | Y | Y | Y | NR | | Y | N | Fair |
| Zhang K | Y | Y | Y | Y | N | NA | Y | NA | Y | N | Y | NR | | Y | N | Poor |
| da Cruz Lage R | Y | Y | Y | Y | N | NA | Y | NA | Y | Y | Y | NR | | Y | Y | Fair |
| Li JX | Y | Y | Y | Y | N | NA | Y | NA | Y | N | Y | NR | | Y | N | Poor |
| Mau | Y | Y | Y | Y | N | NA | Y | NA | Y | Y | Y | NR | | N | Y | Fair |
| Sampaio-Barros PD | Y | Y | Y | Y | N | NA | Y | NA | Y | Y | Y | NR | | N | Y | Fair |
| Yu YX | Y | Y | Y | Y | N | NA | Y | NA | Y | Y | Y | NR | | Y | N | Fair |
| Kumar A | Y | Y | Y | Y | N | NA | Y | NA | Y | N | Y | NR | | N | N | Poor |
| Chen R | Y | Y | Y | Y | N | NA | Y | NA | Y | Y | Y | NR | | Y | N | Fair |
| Xiao ZY | Y | Y | Y | Y | N | NA | Y | NA | Y | Y | Y | NR | | NR | N | Poor |
| ^#^ (1) Defined research question (2) clear study population (3) >50 % participation rate (4) uniform inclusion and exclusion criteria (5) sample size justification (6) exposure of interest measured before outcome (7) sufficient time frame between exposure and outcome (8) examination of different levels of exposure in relation to outcome (9) defined and evenly applied exposure methods (10) exposure assessed more than once over time (11) defined and consistently applied outcome measure (12) blinding of assessors (13) loss of follow-up <20 % (14) key potential confounding variables measured and adjusted statistically for impact between exposure and outcome.  Y, yes; N, no; NA, not applicable; NR, not reported | | | | | | | | | | | | | | | | |
